# Leukocyte capture and modulation of cell-mediated immunity during human sepsis: an *ex vivo *study

**DOI:** 10.1186/cc12587

**Published:** 2013-03-26

**Authors:** Thomas Rimmelé, Ata Murat Kaynar, Joseph N McLaughlin, Jeffery V Bishop, Morgan V Fedorchak, Anan Chuasuwan, Zhiyong Peng, Kai Singbartl, Daniel R Frederick, Lin Zhu, Melinda Carter, William J Federspiel, Adriana Zeevi, John A Kellum

**Affiliations:** 1 The CRISMA (Clinical Research, Investigation, and Systems Modeling of Acute Illness) Center, Department of Critical Care Medicine, University of Pittsburgh, School of Medicine, 3550 Terrace Street, Pittsburgh, PA 15261, USA; 2 McGowan Institute for Regenerative Medicine, University of Pittsburgh, 450 Technology Drive, Pittsburgh, PA 15219, USA; 3 The Thomas E. Starzl Transplantation Institute, University of Pittsburgh, 200 Lothrop Street, Pittsburgh, PA 15261, USA

## Abstract

### Introduction

Promising preclinical results have been obtained with blood purification therapies as adjuvant treatment for sepsis. However, the mechanisms by which these therapies exert beneficial effects remain unclear. Some investigators have suggested that removal of activated leukocytes from the circulation might help ameliorate remote organ injury. We designed an extracorporeal hemoadsorption device capable of capturing both cytokines and leukocytes in order to test the hypothesis that leukocyte capture would alter circulating cytokine profiles and influence immunological cell-cell interactions in whole blood taken from patients with sepsis.

### Methods

We performed a series of *ex vivo *studies in 21 patients with septic shock and 12 healthy volunteers. Blood circulated for four hours in closed loops with four specially designed miniaturized extracorporeal blood purification devices including two different hemoadsorption devices and a hemofilter in order to characterize leukocyte capture and to assess the effects of leukocyte removal on inflammation and immune function.

### Results

Hemoadsorption was selective for removal of activated neutrophils and monocytes. Capture of these cells led to local release of certain cytokines, especially IL-8, and resulted in complex cell-cell interactions involved in cell-mediated immunity. Inhibition of cell adherence reversed the cytokine release and the effects on lymphocyte function.

### Conclusions

Monocyte and neutrophil capture using a sorbent polymer results in upregulation of IL-8 and modulation of cell-mediated immunity. Further studies are needed to understand better these cellular interactions in order to help design better blood purification therapies.

## Introduction

Sepsis is the most common cause of death in the intensive care unit [1]. Care is mostly supportive as specific treatments for sepsis have failed to become universally accepted [2-5]. Blood purification techniques, including high-volume hemofiltration (HVHF), cascade hemofiltration, hemoadsorption, plasmapheresis, coupled plasma filtration adsorption (CPFA), high-adsorption hemofiltration, and high cut-off hemodialysis/hemofiltration, represent a class of therapies for sepsis that have seen promising pre-clinical and early clinical results but have not yet been translated into routine clinical practice [6-12].

There are several explanations for the lack of translation of experimental findings from blood purification studies into clinical practice. First, there are no large multicenter trials evaluating the ability of blood purification therapies to improve patient-centered outcomes in sepsis. Second, the non-specific nature of these therapies in removing inflammatory mediators has raised the concern that small beneficial molecules (for example, antibiotics, nutrients, vitamins, trace elements) may also be removed [13-15]. Third, the mechanisms by which these therapies exert beneficial effects remain poorly understood and controversial [16].

Recent evidence suggests that these therapies may not only remove soluble mediators of inflammation but may also work at the cellular level, modulating immune function by interacting directly or indirectly with inflammatory cells [12,17-20]. Furthermore, adsorptive materials such as polymyxin B not only adsorb inflammatory mediators and toxins but also remove leukocytes due to cell adsorption onto the surface of the blood purification device [21-24]. Some investigators have suggested that removal of activated leukocytes from the circulation could ameliorate remote organ injury [22,23,25]. Apheresis systems have even been developed with the purpose of leukocyte capture in the setting of acute kidney injury, chronic kidney disease and other refractory inflammatory diseases (for example, ulcerative colitis) [25-29].

As part of an ongoing project to develop a blood purification device for the treatment of sepsis, we designed an extracorporeal hemoadsorption device capable of capturing not only cytokines but also leukocytes. We hypothesized that leukocyte capture would alter the immune response to sepsis. To address this, we performed a series of *ex vivo *studies with the aim of characterizing leukocyte capture using different extracorporeal blood purification devices. Two different hemoadsorption devices using sorbent polymer were tested, along with a hemofilter used as a control, and the effects of these interactions on inflammation and immune function were characterized in the setting of human sepsis.

## Materials and methods

### Overview

We obtained blood from both patients with septic shock and healthy volunteers and circulated it for four hours in a closed loop with one of four specially designed miniaturized extracorporeal devices. At baseline and at the end of the session, we measured white blood cell and differential counts, plasma concentrations of various inflammatory mediators, leukocyte cell surface molecule expression and lymphocyte function.

### Subjects

The study was approved by the University of Pittsburgh Institutional Review Board. After consent was obtained, blood was withdrawn from 21 patients with septic shock and from 12 healthy volunteers. Septic shock was defined according to the American College of Chest Physicians/Society of Critical Care Medicine consensus conference criteria [30]. Patients were excluded if pregnant, younger than 18 years, receiving renal replacement therapy, receiving immunosuppressive therapy or if they had a history of hematological malignancy. Healthy volunteers were older than 18 years of age and not pregnant.

### *Ex vivo *circuits and blood purification devices

**Figure 1:**
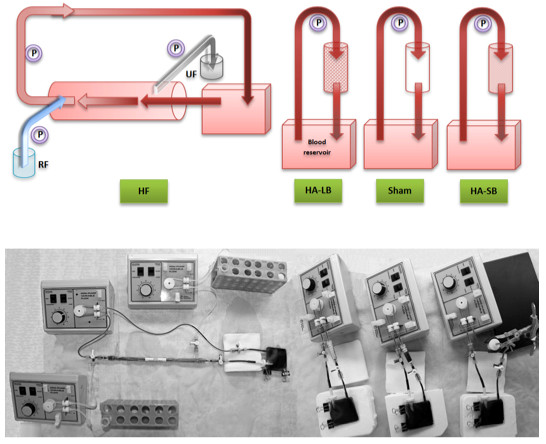
**The four *ex vivo *miniaturized extracorporeal circuits (hemofiltration, hemadsorption with large beads, hemoadsorption with small beads and sham)**. Blood was circulated through these closed circuits for four hours. HA-LB, hemoadsorption with large beads; HA-SB, hemoadsorption with small beads; HF, hemofiltration; P, mini-pump; RF, replacement fluid; UF, ultrafiltrate.

Blood was collected into standard vacuum-collection tubes. The experiments consisted of perfusing blood through four different closed miniaturized *ex vivo *extracorporeal circuits over a period of four hours: 1) a hemoadsorption device containing 1.5 g of large (approximately 550 to 600 μm diameter) polystyrene divinylbenzene copolymer beads (Cytosorb™; CytoSorbents Corporation, Monmouth Junction, NJ, USA); 2) a hemoadsorption circuit containing 2.5 g of small (70 to 75 μm diameter) Cytosorb beads; 3) a hemofiltration circuit with a mini hemofiltration device (Oxiris^®^, Gambro-Hospal, Meyzieu, France); and 4) a sham circuit consisting of the extracorporeal circuit with no blood purification device (that is, a tubing circuit with just an empty cartridge) (Figure 1).

Components of the extracorporeal circuits were polyethylene tubing, a 15 ml blood reservoir (Origen™, Austin, TX, USA), silicone tubing and a peristaltic pump (Control Company, Friendswood, TX, USA) set up with a blood flow rate of 0.75 ml/minute. For the hemofiltration circuit, saline was used for replacement fluid. The ultrafiltration flow rate and the replacement fluid flow rate were both 0.15 ml/minute, maintaining the total blood volume consistently stable at 15 ml. This polyacrilonitrile mini-hemofilter had a surface area of 420 cm^2 ^and was specifically designed and miniaturized for this study. The surface polarity of this blood purification membrane was modified by the addition of a polyethyleneimine coating, a positively-charged polymer, allowing the membrane to catch negatively charged endotoxins via surface adsorption. For the hemoadsorption circuits, the beads were located inside miniaturized cartridges. The difference in diameter between large and small beads resulted in a significant increase of the contact area with blood, from 45 cm^2 ^(large beads) to 297 cm^2 ^(small beads). In all circuits, unfractionated heparin was added to the blood before the beginning of each experiment in order to obtain a heparin concentration of 10 IU/ml. Information regarding the miniaturized hemofiltration and hemoadsorption devices is summarized in Table 1.

**Table 1:** Summary of the characteristics of the miniaturized blood purification devices

	Large bead hemoadsorption device (HA-LB)	Small bead hemoadsorption device (HA-SB)	Hemofiltration device (HF)
Material	Polystyrene divinyl benzene copolymer	Polystyrene divinyl benzene copolymer	Polyacrilonitrile
Average bead diameter (µm)	535	72.5	n/a
Total outer surface area = contact with blood (m^2^)	0.0045	0.0297	0.0420
Total inner porous surface area = contact with plasma (m^2^)	1,275	2,125	Information not provided
Pore size (nm)	< 5	< 5	2.9
Blood flow rate (ml/min)	0.75	0.75	0.75
Ultrafiltrate flow rate (ml/min)			0.15
Replacement fluid flow rate (ml/min)			0.15
Length of the hemofilter fibers (cm)	n/a		21
Hemofilter filter wall thickness (µm)			50
Number of fibers per hemofilter			264
Hemofilter fiber internal diameter (µm)			240

### Electron microscopy and immunofluorescence images

We studied the cell adherence to the hemoadsorption beads using scanning electron microscopy (SEM) and characterized adherent cells using immunofluorescence (IF) microscopy.

#### SEM

Following the experimental period, beads were removed from the cartridges and fixed in 1% OsO4. Specimens dehydrated in alcohol gradients were then imaged using JEOL 9335 SEM.

#### IF

The beads were fixed in 2% paraformaldehyde, blocked with 2% bovine serum albumin (BSA) and then incubated with CD14 (Abcam, Cambridge, MA, USA) primary antibody. Fluor-conjugated phalloidin for F-actin and nuclear stain 4',6-diamidino-2-phenylindole (DAPI) were also co-incubated. Beads were then imaged using an Olympus Fluoview confocal microscope.

### Cytokine measurements

Blood samples were withdrawn from the circuits at baseline and after four hours of circulation. Samples were immediately centrifuged at 1,500 × g for 5 minutes at 4°C and plasma was removed and stored at -80°C until assayed. Granulocyte macrophage colony-stimulating factor (GM-CSF), tumor necrosis factor (TNF), interferon γ and the interleukin (IL) -1 β, IL-2, IL-4, IL-5, IL-6, IL-8, and IL-10 concentrations were measured in duplicate by Luminex^® ^bead technology (Invitrogen, Camarillo, CA, USA).

### Leukocyte surface marker expression

Cell surface markers were measured in monocytes, neutrophils and lymphocytes. Blood samples were withdrawn at baseline and after four hours of circulation through the circuits. Erythrocytes were lysed with BD Pharm Lyse™ solution (Becton Dickinson, Franklin Lakes, NJ, USA) and washed in 1% BSA in PBS. Fc receptors were blocked with excess immunoglobulin G (IgG). Cells stained for surface markers were incubated with appropriate antibodies (Becton Dickinson) and fixed in 1% paraformaldehyde. For nuclear factor kappa B (NFκB), cells were treated with Cycletest Plus Kit (Becton Dickinson) prior to staining with anti-NFκB antibody (Santa Cruz Biotechnology Inc, Santa Cruz, CA, USA). Data were acquired on a Beckman Coulter XL-MCL and analyzed with FCS Express (De Novo Software, Los Angeles, CA, USA). Cells were gated by size and granularity for neutrophils and monocytes. NFκB cells were gated for positive staining of propidium iodide (PI) and by size. HLA-DR, CD 11b, CD 11a, CD 62L, TNF-alpha converting enzyme (TACE), and NFκB expression was measured by the geometric mean of fluorescence intensity. Apoptosis was measured as percent positive for Annexin V with PI excluding necrotic cells. T-cells were stained with BD Fast-Immune mix of CD3/CD4/CD69. T-Lymphocytes were gated on CD3+ and gated into CD4+ and CD4- populations. CD69 was measured as % positive and as geometric mean of CD3+/CD4+ and CD3+/CD4- cells.

### Measurement of lymphocyte function

We measured intracellular ATP production in CD3+ and CD4+ lymphocytes as a marker of global lymphocyte immune function following phytohemagglutinin (PHA) and concavalin (Con-A) mitogen stimulation (The Cylex's Immuknow^® ^& T-Cell Memory™ assays, Cylex Inc., Columbia, MD, USA). Briefly, for both assays, 250 μl of whole blood was diluted with sample diluent, then added to the appropriate wells of the microtiter plates and incubated for 15 to 18 hours at 37°C, with and without stimulant (PHA or Con-A). The following day, CD4+ or CD3+ cells were selected within the microwells using magnetic particles coated with anti-human CD4 and CD3 monoclonal antibodies (Dynabeads^®^, Dynal Biotech ASA, Oslo, Norway), washed to remove residual cells and lysed with lysis reagent to release intracellular ATP. ATP was measured using a luciferase-based reaction with a luminometer. Results were expressed as the amount of ATP in nanograms per milliliter of whole blood.

### Experiments with ethylenediaminetetraacetic acid

Blood was withdrawn from seven healthy volunteers and was circulated through the four circuits during four hours. Blood collection tubes containing ethylenediaminetetraacetic acid (EDTA) were used and no additional anticoagulation was added in the circuits after blood draw, leading to an EDTA concentration of 1.8 mg per milliliter of blood. White blood cell and differential counts, cytokine level measurements and lymphocyte function activity were recorded.

### Statistical analysis

Non-normally distributed continuous data were expressed as the median and interquartile range (IQR). Where indicated, values were normalized to conditions before exposure to the indicated devices and are reported as % Uncirculated. Comparison of group differences for continuous variables was done by the non parametric Mann-Whitney test. Statistical tests were performed using the Sham group as the reference. A statistical and graphing software program (GraphPad Prism, version 5, GraphPad Software, La Jolla, CA) was used for data processing and analysis. All *P *values were two-sided and statistical significance was set at an α-value of 0.05.

## Results

### Hemoadsorption devices were selective for removal of neutophils and monocytes

**Figure 2:**
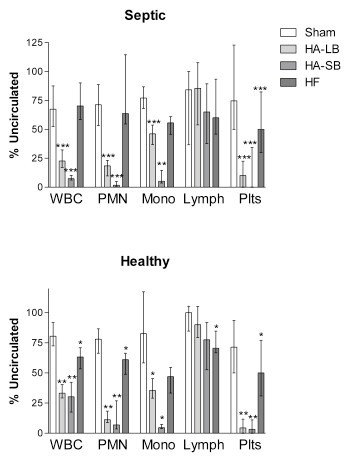
**Effects of extracorporeal circuits on white blood cells (WBC), neutrophils (PMN), monocytes (Mono), lymphocytes (Lymph) and platelets (Plts)**. Results are expressed as % of cells present before circulation through the circuits (uncirculated condition), in blood obtained from septic patients and healthy volunteers (medians with interquartile ranges). *P < .05, **P < .01, ***P < .001 compared with Sham.

**Figure 3:**
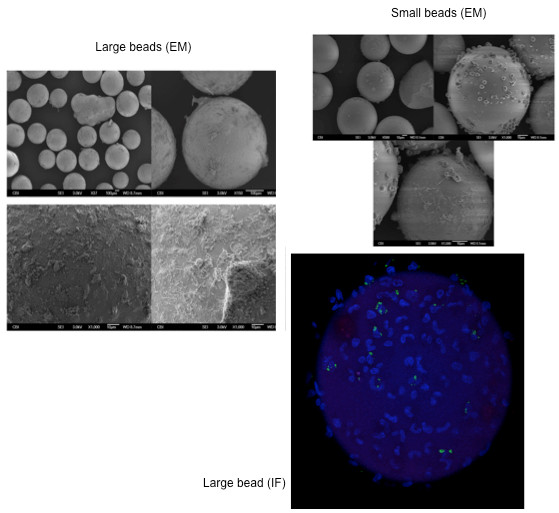
**Electron microscopy (EM) and immunofluorescence (IF) images showing cell adsorption onto the beads**.

Blood circulation through all four circuits resulted in the removal of leukocytes and platelets, both from septic and healthy blood (Figure 2). This was particularly significant for hemoadsorption, especially with the small beads, where less than 10% of platelets and leukocytes remained circulating at the end of the experiments with septic blood. Importantly, the leukocyte depletion was primarily seen in monocytes and neutrophils whereas lymphocytes showed little affect. The circuit without a blood purification device (sham circuit) was responsible for a slight decrease in leukocytes and platelets. EM and IF images confirmed adsorption of leukocytes and platelets onto the surface of the beads (Figure 3).

### Hemoadsorption resulted in depletion of CD11b positive cells and led to release of IL-8 and TNF

**Figure 4:**
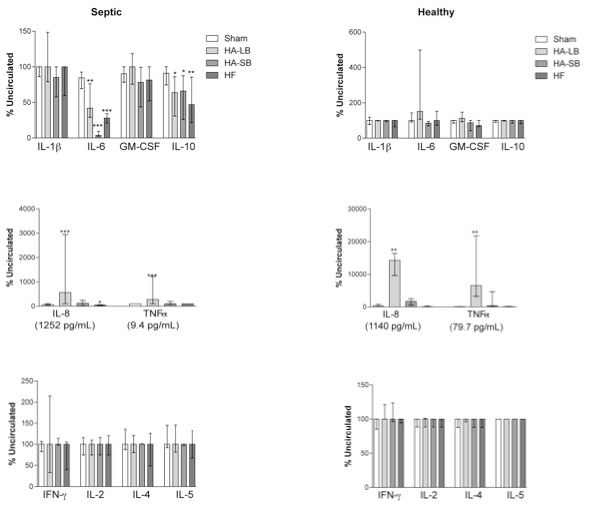
**Effects of extracorporeal circuits on cytokine concentrations, in septic and healthy blood**. Data are expressed as % of the uncirculated condition (medians with interquartile ranges). GM-CSF, granulocyte macrophage colony stimulating factor; IFN, interferon; IL, interleukin; TNF, tumor necrosis factor. *P < .05, **P < .01, ***P < .001 compared with Sham.

**Figure 5:**
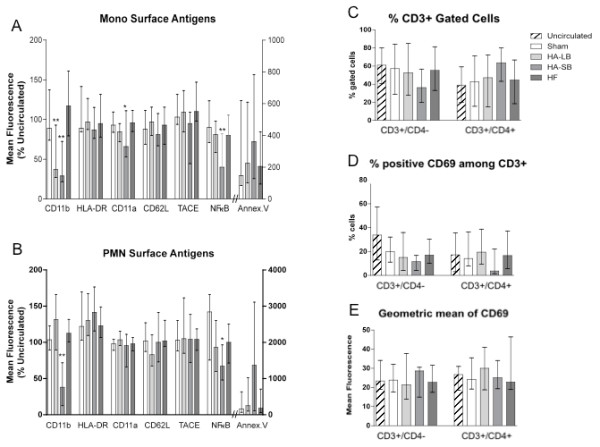
**Effects of extracorporeal circuits on leukocyte surface marker expression, in septic blood**. (**A, B) **Geometric mean of fluorescence intensity of CD 11b, HLA-DR, CD 11a, CD 62L, TACE, and NFkB and positive cells for Annexin V in monocytes and PMN. Data are expressed as % of the uncirculated condition (medians with interquartile ranges). (**C) **Percentage of CD3+/CD4- and CD3+/CD4+ cells among T-lymphocytes (medians with interquartile ranges). (**D) **Percentage of positive cells for CD69 among CD3+/CD4- and CD3+/CD4+ cells (medians with interquartile ranges). (**E)**: Geometric mean of fluorescence intensity of CD69 in CD3+/CD4- and CD3+/CD4+ cells (medians with interquartile ranges). *P < .05, **P < .01, ***P < .001 compared with Sham. Annex. V, Annexin V; Mono, monocytes; NFkB, nuclear factor-kappa B; PMN, neutrophils; TACE, tumor necrosis factor-alpha converting enzyme.

Compared to baseline, hemofiltration and hemoadsorption decreased the concentration of IL-6 and IL-10 in septic blood (Figure 4). As expected, cytokine levels in healthy volunteers were very low. A significant production of IL-8 and TNF occurred with the large bead hemoadsorption device; levels increased in both septic and healthy blood. Although changes in TNF were statistically significant, levels were quite low, especially in septic blood (< 10 pg/ml). However, IL-8 levels increased dramatically, exceeding 1,000 pg/ml in both septic and healthy blood. IFN-γ, IL-2, IL-4 and IL-5 levels did not change after circulation through the devices. Neutrophils and monocytes remaining in circulation at the end of the experiment with hemoadsorption were less likely to express CD11b (Figure 5). By contrast, molecules expressed on the surface of lymphocytes were not changed after blood purification using the different devices.

### Monocyte depletion leads to modulation of lymphocyte function

**Figure 6:**
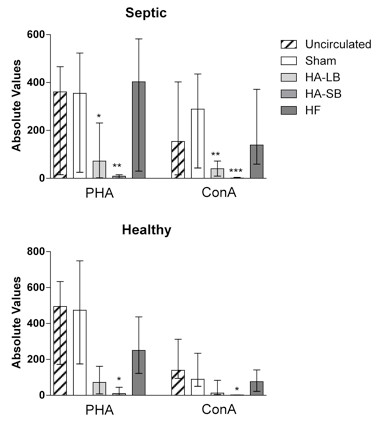
**Effects of extracorporeal circuits on Immuknow & T-Cell Memory assays in septic and healthy blood**. Data are expressed as absolute values of the amount of ATP in nanograms per milliliter of whole blood (medians with interquartile ranges). *P < .05, **P < .01, ***P < .001 compared with Sham. Con-A, concavalin A; PHA, phytohemagglutinin.

Although lymphocytes were not captured by the devices, T cell activity (both CD3+ and CD4+) was decreased with hemoadsorption, both in septic and healthy blood (Figure 6). In septic blood, the Immuknow value with PHA stimulation decreased from 361 (53 to 462) to 72 (3 to 219), *P *= 0.0464 (large beads), and to 9 (2 to 13), *P *= 0.0048 (small beads). Similar results were seen with Con A stimulation: 154 (17 to 394) to 40 (15 to 69), *P *= 0.0071 (large beads) and to 1 (0.3 to 3) *P *= 0.0007 (small beads). By contrast, there was no significant change of the Immuknow value when blood was circulated through the sham and the hemofiltration circuits.

### EDTA blocked cell capture, ameliorated IL-8 release and reversed T-cell suppression

**Figure 7:**
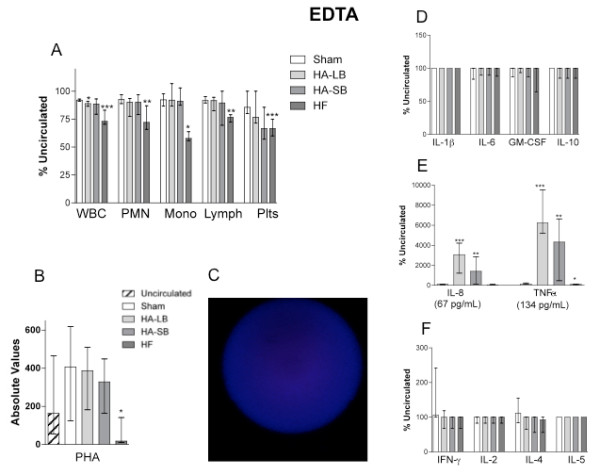
**Experiments with EDTA**. Effects of extracorporeal circuits on WBC count (**A**), cytokine concentrations (**D, E, F**) and Immuknow & T-Cell Memory assays (**B**), in healthy blood, when circuit anticoagulation was performed with EDTA instead of heparin. Plots show the medians with interquartile ranges. An immunofluorescence picture of a large bead after blood circulation is also shown (**C**). *P < .05, **P < .01, ***P < .001 compared with Sham. EDTA, ethylenediaminetetraacetic acid; WBC, white blood cell.

In order to determine if the effects on cytokines and T-cells were mediated by capture of neutrophils and/or monocytes, we circulated healthy blood through the circuits after anticoagulation with increased concentrations of heparin, citrate and EDTA. In these experiments, there was no change with heparin and citrate (data not shown). However, EDTA blocked leukocyte and platelet capture (Figure 7) leading to markedly attenuated IL-8 release (reaching only 67 pg/ml with the large bead device). Immuknow values remained similar with hemoadsorption as compared to the baseline value. Of note, the Immuknow is affected by EDTA since the PHA results before circulation through the circuits are already reduced (see comparison with Figure 6). Con A is suppressed completely and cannot be evaluated (data not shown).

## Discussion

In this *ex vivo *study using human whole blood taken from patients with sepsis and healthy volunteers, we tested the hypothesis that leukocyte capture modulates inflammatory cytokines and immune cell function. We designed a new hemoadsorption device, capable of capturing not only inflammatory mediators but also activated leukocytes, and we examined the effects of this therapy on inflammation and immune function. Our results showed that while monocytes and neutrophils (along with platelets) were captured by the hemoadsorption device, lymphocytes were not affected. Furthermore, capture was preferential to activated cells as evidenced by the marked reduction in CD11b+ and less dramatic reduction in CD11a+ cells remaining in circulation after treatment. Importantly, capture of monocytes and neutrophils led to local release of certain cytokines, especially IL-8, and resulted in changes in T-cell function as measured by response to stimulation with PHA and Con A. Inhibition of monocyte and neutrophil capture with EDTA nearly abolished IL-8 release and reversed the T cell suppression.

Numerous experimental and clinical studies have reported that various forms of extracorporeal blood purification used in sepsis are effective in clearing inflammatory mediators and endotoxins from the plasma and are responsible for an improvement of physiological parameters (for example, hemodynamics, oxygenation) [7,8,10,17,31]. Cruz *et al*. recently showed reduced mortality when hemoadsorption was used in patients with abdominal sepsis [2]. However, the mechanisms by which these therapies exert beneficial effects are not entirely understood [16]. Recent evidence suggests that these blood purification therapies may not only remove soluble mediators of inflammation but may also work at the cellular level, modulating the immune function by interacting directly or indirectly with inflammatory cells [12,17-20]. Moreover, it has recently been shown that polymyxin B hemoadsorption does more than adsorb inflammatory mediators and toxins-- it also removes leukocytes via cell adsorption onto the surface of the device [21-23]. Kumagai *et al*. found very similar results to ours with a significant decrease of neutrophils, monocytes and lymphocytes after *ex vivo *polymyxin B hemoadsorption (78%, 70% and 10%, respectively, compared with baseline values). Additionally, this study also suggested that the adhesion of leukocytes preferentially affected activated neutrophils, therefore resulting in a decreased ability for the remaining circulating cells to cause endothelial damage [22]. Similarly, Abe *et al*. also demonstrated that a large number of cells captured by polymyxin B-immobilized fiber columns were activated neutrophils, highly expressing HLA-DR, CD14, CD62L and CD114 [21]. Finally, another recent report from Nishibori *et al*. also suggested that a polymyxin B-immobilized fiber column specifically removed monocytes that had been activated during septic shock [23].

In our study, when blood was exposed to the hemoadsorption device, we observed that circulating platelet, monocyte and neutrophil counts decreased due to their adsorption onto the beads (Figures 2 and 3). Leukocyte and platelet counts were both significantly reduced with hemoadsorption compared to sham and were even more reduced with the small beads, as compared to the large beads, most likely due to the increased surface area and reduced interstitial space between beads in the device. Moreover, it seems likely that adsorption of platelets, neutrophils and monocytes occurred in an inter-related way [32,33]. Indeed, it is known that platelets are readily activated by shear force and contact with biomaterials [34]. This activation, in turn, leads to changes in the surface expression of integrins and adhesion molecules, leading to platelet aggregation and deposition on biomaterials. Platelet activation also leads to release of cytokines, thereby providing both the chemical signal and appropriate surface for leukocyte activation and adhesion [35,36].

Importantly, we also found that captured leukocytes released low levels of TNF and very high levels of IL-8. IL-8, a chemokine, is mainly produced by activated monocytes and functions to recruit neutrophils (IL-8 is also known as neutrophil chemotactic factor) [37]. Interestingly, our devices did not capture lymphocytes. Lymphocyte concentrations remained quite stable after circulation through the circuits and this correlates with the absence of production of the lymphokines IL-2, IL-4, IL-5 and IFNγ (Figure 4) and the absence of changes in the expression of CD4, CD8 and CD69 on the lymphocyte surface (Figure 5).

We evaluated T-lymphocyte function with the Immuknow test. This assay is approved for monitoring immune cell function in immunosuppressed populations [38]. Immune cell function is considered low when values of this assay are below 225 ng/ml, medium when 225 to 525 ng/ml and high (normal) when more than 525 ng/ml [39]. T-cell function was much more affected with small bead hemoadsorption rather than with large beads. Indeed, after four hours of circulation in the small bead circuits, lymphocytes were unresponsive (Figure 6). Furthermore, our findings support the conclusion that lymphocyte function was not altered because of a decrease in the number of lymphocytes or lymphocyte subtypes (Figures 2 and 5). Since the activation of T cells needs to occur in the presence of antigen presenting cells, the removal of monocytes that function as antigen presenting cells in this *in vitro *system most likely explains the absence of response from the T-cells. This was confirmed by the EDTA experiments that abolished cell capture and completely reversed the inhibition of lymphocyte activation.

Our study has some limitations. First, as an *ex vivo *investigation, we cannot determine the clinical effects of the changes in cell numbers and function we observed. Our closed loop experimental design using fresh whole blood *ex vivo *is not comparable to that seen *in vivo*. Indeed, *ex vivo *circuits work as a single compartment model, whereas the *in vivo *use of a hemoadsorption cartridge integrates into a multi-compartment state where activated cells move into tissues or adhere to vessel walls and are then not available for capture by the device. Moreover, as compared to the total blood volume of the *ex vivo *circuit, the surface area of the hemofiltration membrane was proportionally larger than what it could be in a human *in vivo *condition. The effects of the devices could, therefore, have been magnified. Second, our results on leukocyte surface marker expression reflect only the remaining circulating cells not those adsorbed onto the beads. Indeed, we could not study the adherent cells directly and can, therefore, only infer what their surface marker expression profile was like. Moreover, we are unable to answer the question whether or not the cell adsorption was a transient or a definitive phenomenon. Third, the relatively low Immuknow absolute values observed in all groups during the EDTA experiments (particularly with Con A), as compared with heparin or citrate anticoagulation, may most likely be explained by the metal ion chelating properties of EDTA, affecting peripheral blood mononuclear cell proliferation and stimulation [40]. Indeed, we were surprised to observe no change in cell capture with heparin or citrate despite some evidence that these agents have differing effects when used for anticoagulation of extracorporeal devices in models of sepsis [41].

These limitations aside, this study supports the novel concept that capture of activated leukocytes from the circulation can be achieved by hemoadsorption. Such therapy could be beneficial by removing activated cells that have deleterious effects, such as disruption of endothelial integrity and impairment of microcirculation by overproduction of proteases and oxygen radicals [22,42-44]. This approach is already used as a therapeutic strategy for other inflammatory diseases. Indeed, some extracorporeal apheresis systems (for example, cellulose acetate beads) have been developed to remove activated monocytes and granulocytes in order to treat specific refractory and inflammatory diseases associated with leukocytes such as ulcerative colitis, neutrophilic dermatosis and psoriatic arthritis [27-29]. More recently, Humes *et al*. have also developed a selective cytopheretic inhibitory synthetic membrane to treat the immunological dysregulation of acute kidney injury and chronic kidney disease, binding and inhibiting circulating activated leukocytes along a continuous renal replacement extracorporeal circuit [26,45].

## Conclusions

In summary, our study shows that capture of activated monocytes and neutrophils by hemoadsorption interferes with the immune response, modulating T-cell function. These findings lead us to propose a new mechanism for how blood purification therapies may modulate the immune response in patients with sepsis. Additional studies are needed to further investigate this concept.

## Key messages

• Leukocyte capture using a hemoadsorption device modulates cell-mediated immunity.

• Removal of activated leukocytes may offer great potential as an adjunct treatment for sepsis and may help explain some of the beneficial effects of existing blood purification therapies.

• Further knowledge of the complex cell-cell interactions that occur inside a leukocyte capture device is needed in order to develop this technology.

## Abbreviations

BSA: bovine serum albumin; Con-A: concavalin-A; CPFA: coupled plasma filtration adsorption; EDTA: ethylenediaminetetraacetic acid; GM-CSF: granulocyte macrophage colony-stimulating factor; HVHF: high-volume hemofiltration; IF: immunofluorescence; IL: interleukin; IQR: interquartile range; PHA: phytohemagglutinin; PI: propidium iodide; PBS: phosphate buffered saline; SEM: scanning electron microscopy; TNF: tumor necrosis factor.

## Competing interests

TR has received consulting fees from Gambro. KS has received speaker honorarium from Cytosorbents. JK has received consulting fees from Cytosorbents, Gambro and Spectral Diagnostics and has also received research grants from Cytosorbents, Gambro, Kaneka, and Spectral Diagnostics. JK has licensed unrelated technologies through the University of Pittsburgh to Cytosorbents and Spectral Diagnostics. None of the other authors report any competing interests.

## Authors' contributions

TR, AMK and JAK participated in research design, writing of the manuscript, performance of the research, and data analysis; JB, MF, AC, ZP, KS, DRF, LZ, MC, WJF and AZ participated in performance of the research and data analysis; JNM participated in statistical aspects of data analysis; all authors contributed to the critical review and revision of the manuscript and approved it for publication.
